# Components of the Lectin Pathway of Complement in Haematologic Malignancies

**DOI:** 10.3390/cancers12071792

**Published:** 2020-07-04

**Authors:** Maciej Cedzyński, Anna S. Świerzko

**Affiliations:** Laboratory of Immunobiology of Infections, Institute of Medical Biology, Polish Academy of Sciences, Lodowa 106, 92-232 Łódź, Poland; aswierzko@cbm.pan.pl

**Keywords:** cancer, collectin, complement, ficolin, haematopoietic stem cells transplantation (HSCT), infection, lectin pathway, MBL-associated serine protease (MASP)

## Abstract

The complement system is activated cascadically via three distinct major routes: classical pathway (CP), alternative pathway (AP) or lectin pathway (LP). The unique factors associated with the latter are collectins (mannose-binding lectin, collectin-10, collectin-11), ficolins (ficolin-1, ficolin-2, ficolin-3) and proteins of the mannose-binding lectin-associated serine protease (MASP) family (MASP-1, MASP-2, MASP-3, MAp19, MAp44). Collectins and ficolins are both pattern-recognising molecules (PRM), reactive against pathogen-associated molecular patterns (PAMP) or danger-associated molecular patterns (DAMP). The MASP family proteins were first discovered as complexes with mannose-binding lectin (MBL) and therefore named MBL-associated serine proteases, but later, they were found to interact with ficolins, and later still, collectin-10 and collectin-11. As well as proteolytic enzymes (MASP-1, MASP-2, MASP-3), the group includes non-enzymatic factors (MAp19, MAp44). In this review, the association-specific factors of the lectin pathway with haematologic malignancies and related infections are discussed.

## 1. Factors Specific for Activation of the Complement System via the Lectin Pathway

**Figure 1:**
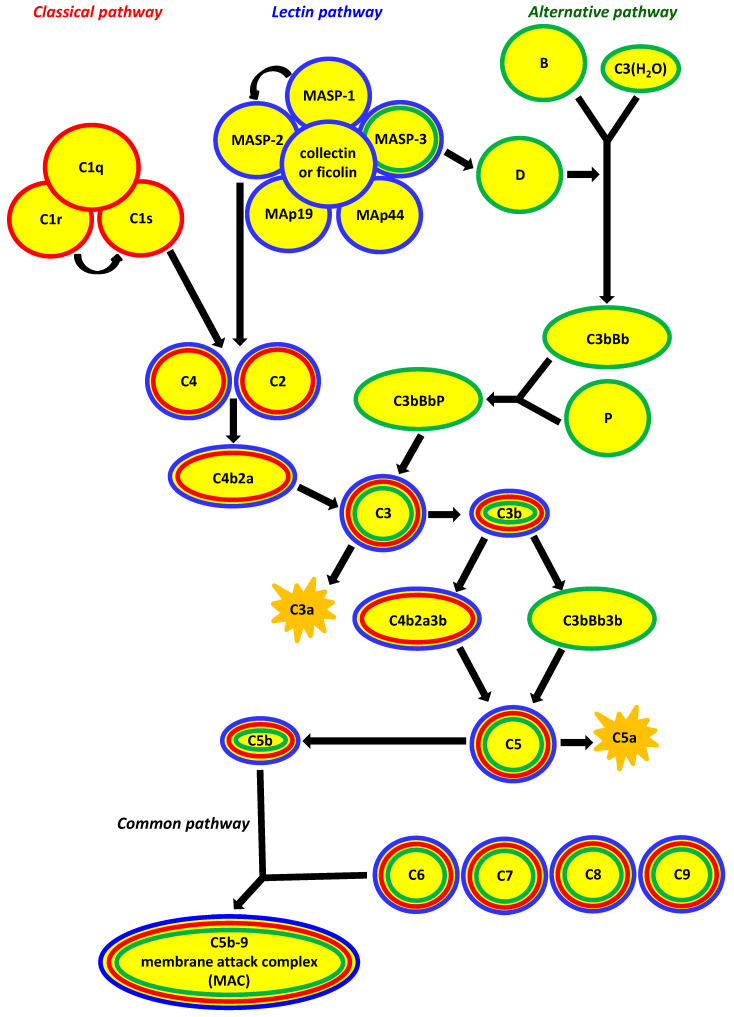
The three major pathways of complement activation. They differ crucially in their initiating events: the classical pathway depends on antibody recognition and binding to C1q; the alternative pathway depends on low-level spontaneous hydrolysis of C3 being stabilised by bacterial polysaccharides, etc.; and the lectin pathway depends on the recognition of polysaccharides/glycoconjugates by collectins (mannose-binding lectin (MBL), CL-10, CL-11) and ficolins (ficolin-1, ficolin-2, ficolin-3). The common result is the generation of C3b from C3; the classical and lectin pathways produce C4b2a as the C3 convertase, whereas that role is played by C3bBb in the alternative pathway. Consequently, C4b2a3b and C3bBb3b are C5 convertases for classical/lectin and alternative pathways, respectively. Finally, membrane attack complex (MAC, C5b-9) incorporates into target cell membrane (common pathway). MAp44 and MAp19 (MBL-associated proteins, 44 kDA and 19 kDa) are non-enzymatic alternative splicing products of *MASP1/3* and *MASP2* genes, respectively, acting as regulators of the lectin pathway. Other regulators, for example C1-inhibitor, C4bp (classical and lectin pathways) or H factor (alternative pathway) have been omitted to simplify the scheme (according to [3], modified).

The complement system is a major branch of the immune response, cross-talking with a variety of immune mechanisms, both innate and acquired. It is activated cascadically via three distinct major routes: the classical pathway (CP), lectin pathway (LP) or alternative pathway (AP). Although each is differentially initiated and characterised by unique, specific factors, all include the activation of C3 and C5 and lead to a common pathway, resulting in the formation of the membrane attack complex (MAC, C5b-9), which binds to the target cell membrane, resulting in cell lysis (Figure 1). The unique factors associated with LP activation are collectins (mannose-binding lectin, collectin-10, collectin-11), ficolins (ficolin-1, ficolin-2, ficolin-3) and proteins of the mannose-binding lectin-associated serine protease (MASP) family (MASP-1, MASP-2, MASP-3, MAp19, MAp44). Both collectins and ficolins (shortly characterised in Table 1) are pattern-recognising molecules (PRM), reactive against pathogen-associated molecular patterns (PAMP) or danger-associated molecular patterns (DAMP). The latter term includes endogenous ligands, for example, aberrantly glycosylated host cell surface structures. Despite the complement activation-dependent direct lysis of pathogen/abnormal self-cells, those factors act as opsonins, and thus contribute to phagocytosis. The MASP family proteins were first described in complexes with mannose-binding lectin (MBL), and therefore named MBL-associated serine proteases but later they were found to interact with ficolins, and later still with collectin-10 and collectin-11. As well as proteolytic enzymes (MASP-1, MASP-2, MASP-3) (Table 1), that group includes also non-enzymatic factors (MAp19, MAp44) [1,2,3,4].

## 2. Associations of the Complement System with Cancer

The role of the complement system in cancer is complex. Its activation may contribute to both the inhibition and progression of tumour growth, and even to metastasis. Some complement factors are considered to be biomarkers contributing to diagnosis or efficacy of therapeutic intervention. Complement is involved in the elimination of apoptotic/necrotic/cancer cells and some carcinogenic pathogens, thus contributing to the prevention of tumourigenesis [21,22,23,24,25,26]. On the other hand, complement-associated chronic inflammation may favour the transformation of host cells, and sublytic complement activation may disturb cell signalling, promote cell proliferation and activate proto-oncogenes [26,27,28,29,30,31,32,33]. Anaphylatoxins (C3a, C5a) were demonstrated to induce epithelial-mesenchymal transformation (EMT), activate matrix metalloproteinases and suppress the function of immune cells in the tumour microenvironment [26,30,31,32,34,35,36,37,38,39]. C5a is thought to contribute to angiogenesis [39]. Furthermore, complements affect the patient response to chemotherapy and contribute to the mobilization of haematopoietic cells from bone marrow to peripheral blood [40,41,42,43].

## 3. Haematologic Malignancies

Stem cells have to function to keep homeostasis and prevent tissue atrophy or aplasia. Haematopoietic stem cells (HSC) are able to differentiate into a variety of mature blood cells of several distinct lineages. That process involves intermediate progenitor cells characterized by gradually lowered differentiation potential. The precisely regulated balance between HSC self-renewal and maturation ensures the production of normal blood cells. The dysregulation of haematopoiesis may result in numerous diseases differing in cell lineage and clinical manifestations, collectively termed haematological malignancies or blood cancers. They are generally divided into 2 basic groups; myeloid and lymphoid malignancies. Both include aggressive, often rapidly fatal disorders, as well as chronic diseases. The first group comprises acute myeloid leukaemia, myeloproliferative neoplasms (including chronic myeloid leukaemia) and myelodysplastic syndromes (including refractory cytopenia with multilineage dysplasia). The second group comprises precursor lymphoid neoplasms (including B- and T-lymphoblastic leukaemias and lymphomas), mature B-cell neoplasms (multiple myeloma, diffuse large B-cell lymphoma, follicular lymphoma and numerous others), and Hodgkin’s lymphoma [44,45,46,47]. Haematological malignancies often compromise the immune defence, as well as bone marrow function in the case of its infiltrations by malignant cells. Intensive chemotherapy and/or radiotherapy add further immunosuppression (mostly due to profound and prolonged neutropenia) [48,49].

## 4. Lectin Pathway-Related Pattern-Recognising Molecules

### 4.1. Collectins

Mannose-binding lectin (MBL), known also as mannan-binding lectin and mannose-/mannan-binding protein (MBP), is a multimer of basic triplet subunits, consisting of identical polypeptide chains. Its molecule is characterized by four regions: an N-terminal cysteine-rich domain, a collagen-like domain, an α-helical neck region and a C-terminal carbohydrate-recognition domain (reviewed by Thiel and Gadjeva [2] and Cedzyński et al. [5]).

MBL, in association with Ca^2+^ cations, recognizes residues of such carbohydrates as D-mannose (D-Man), N-acetyl-D-glucosamine (D-GlcNAc) or L-fucose (L-Fuc). This enables it to interact with numerous microbial polysaccharides or glycoconjugates like capsular polysaccharides, lipopolysaccharides, fungal mannans, etc. Furthermore, it also binds phospholipids and nucleic acids [5]. It should be stressed that MBL is able to recognise not only a variety of pathogens, but also senescent fibroblasts [50], late apoptotic and necrotic cells [51] and some cancer cells carrying aberrantly glycosylated surface structures [52].

Single nucleotide polymorphisms (SNP) of the *MBL2* gene promoter region: −550 G > C (rs11003125, usually called H/L) and −221 C > G (rs7096206, Y/X) influence MBL serum concentration. Coding region SNP: +223 C > T (Arg52Cys, rs5030737), +230 G > A (Gly54Asp, rs1800450) and +239 G > A (Gly57Glu, rs1800451), known as A > D, A > B and A > C, respectively (their variant alleles are collectively designated O), affect both MBL level and activity. The presence of O alleles is associated with diminished opsonic properties and complement activation, due to the impaired oligomerization of the molecule and the ability to form complexes with MASP. The increased sensitivity to endogenous metalloproteases contributes, in turn, to a lower serum MBL concentration. Strong linkage disequilibria exist between the aforementioned SNP [and additionally the +4 C > T (rs7095891, P/Q, in *MBL2* gene exon 1 5′-untranslated region)]; only seven haplotypes (giving 28 genotypes) are considered relatively common: HYPA, LYPA, LYQA, LXPA, HYPD, LYPB, LYQC. Other (rare) haplotypes reported are LYPD, LYQB, HXPA, and HYPB. MBL primary deficiency, believed to be the commonest human immunodeficiency (affecting 5–10% of the population) is associated with LXA/O and O/O genotypes (reviewed by Cedzyński et al. [5]) Furthermore, several polymorphisms located within the 3′-untranslated region (exon 4) were reported to influence MBL serum concentration (Ex4-710 A > G (rs2099902), Ex4-901 A > G (rs2120132), Ex4-1047 T > G (rs12254577), Ex4-1067 G > A (rs10824792), Ex4-1483 T > C (rs10082466)) [53].

Two other collectins able to activate complements via the lectin pathway, collectin-10 (CL-10, known also as collectin liver-1, CL-L1) and collectin-11 (CL-11 or collectin kidney-1, CL-K1) were found to form heterooligomers, termed CL-LK [6,7,54]. Hansen et al. [7] reported the widespread tissue distribution of both CL-10 and CL-11, and their high expression in endo-/exocrine secretory tissues and mucosa.

Collectin-11 recognizes such carbohydrate ligands as D-mannose (D-Man), L-fucose (L-Fuc) and N-acetyl-D-mannosamine (D-ManAc), in the presence of calcium ions. As well as the ability to interact with surface structures of some Gram-negative and Gram-positive bacteria, fungi and influenza A virus [6,9,10,55], it was found to bind to DNA of various origins and suspected to contribute to the response to apoptotic cells, neutrophil extracellular traps and biofilms [56]. Furthermore, CL-11 was supposed to contribute to complement-mediated ischaemic injury via interaction with L-fucose at the site of ischaemic stress [57,58].

CL-10 is able to recognise D-mannose (D-Man), N-acetyl-D-glucosamine (D-GlcNAc), D-galactose (D-Gal), D-fucose (D-Fuc) and L-fucose (L-Fuc). Although the microbial/abnormal self-structures targeted by that collectin have not been identified, as a component of CL-LK heterooligomers, it may broaden its range of interactions and/or modify their affinity [7,8].

Several *COLEC11* polymorphisms, were reported to influence CL-11/CL-LK serum concentration (promoter region SNP −9570 C > T, rs3820897) or its activity (exon 7, encoding for lectin domain, +39618 C > G, His219Arg, rs7567833) [59]. Furthermore, the variant homozygosity for +610 G > A (Gly204Ser, rs387907076) SNP was found to be associated with CL-11 deficiency [60]. That polymorphism, together with some others: +496 T > C (Ser169Pro, rs387907075), single nucleotide deletions (+45delC, (RCV000023960), +300delT, both resulting in frame-shift and pre-mature termination), in-frame deletion (+648.650delCTC, (RCV000023962) causing a loss of a serine residue at position 217), and a huge (27-kb) deletion (complete loss of the N-terminal and partial of collagen-like domains) were moreover found to contribute to development of a rare autosomal disorder, 3MC syndrome [61]. Later, those mutations were found to prevent CL-11 secretion, probably due to the disruption of calcium cations binding during its biosynthesis [62].

Among *COLEC10* gene promoter polymorphisms, the deletion of five nucleotides (−161/−157AAAATdel, rs148350292) was suggested to disturb the binding of several transcription factors essential for liver development or immune response modulation. Furthermore, the +3654 C > T SNP (Arg125Trp, rs149331285) localised to exon 5 affects the protein structure as well as the protein level in blood (significantly higher CL-LK serum concentrations were found in heterozygotes, compared with C/C homozygotes) [59]. Later, Munye et al. [63] reported other *COLEC10* coding region mutations: +25 C > T (Arg9Ter), +226delA (Gly77Glufs*66), supposed to be associated with the synthesis of truncated protein or non-sense mediated decay. Another mutation, +528 C > G (Cys176Trp), was predicted to damage the structure of the CL-10 lectin domain. The three mutations mentioned were found in the 3MC syndrome-affected family [63].

### 4.2. Ficolins

Ficolins, like collectins, are oligomeric, collagen-related pattern recognising molecules. Although their structure and activity are generally similar to those of collectins, they possess no typical lectin (carbohydrate-binding) domain, but a C-terminal fibrinogen-like region, responsible for interactions with target structures. Ficolins are known to bind to acetyl (not hydroxyl, as typical lectins) groups, but are not necessarily present in sugar residues [1,2,4].

Although expressed in various cell types (ficolin-1 in bone marrow, monocytes and neutrophils; ficolin-2 in hepatocytes; ficolin-3 in hepatocytes, alveolar type II pneumocytes and ciliated bronchial cells), all ficolins circulate in blood and participate in the systemic immune response. Ficolin-1, present in lung macrophages, and ficolin-3 are able to act locally as well, in the respiratory system (reviewed by Matsushita et al. [4] and Matsushita [11]).

Ficolin-1, known also as M-ficolin, recognizes such ligands as N-acetyl-D-glucosamine (D-GlcNAc), N-acetyl-D-mannosamine (D-ManNAc), N-acetyl-D-galactosamine (D-GalNAc) and sialic acid [11]. As well as microbial surface structures (like bacterial capsular polysaccharides), it binds to mitochondria from damaged host cells and (via complexing with pentraxin-3) apoptotic/necrotic cells, which is important for maintaining homeostasis [64,65]. It should be stressed that sialic acid-rich glycoproteins are often over-expressed on the surface of metastatic cancer cells.

The *FCN1* gene encoding for ficolin-1 is highly polymorphic. The variant (A) alleles corresponding to −542 G > A (rs10120023) and −144 C > A (rs10117466) SNP are associated with higher gene expression, and therefore higher ficolin-1 serum concentration. In contrast, the minority alleles related to +6658 G > A (Ala218Thr, rs148649884), +7895 T > C (Ser268Pro, rs150625869) and +7959 A > G (Asn289Ser, rs138055828) polymorphisms were found to be responsible for lower ficolin-1 levels. Furthermore, the presence of threonine at position 218 and serine at position 289 affects its pattern recognition properties [66,67,68]. Although no case of ficolin-1 deficiency has been reported, it is suspected that variant homozygotes for +7895 T > C SNP would be totally deficient [67].

Ficolin-2 (or L-ficolin), in contrast to other ficolins, possesses four binding sites in its fibrinogen (FBG) domain that enable it to recognize a broad range of ligands (N-acetyl-D-glucosamine (D-GlcNAc), N-acetyl-D-galactosamine (D-GalNAc), N-acetyl-D-mannosamine (D-ManNAc), D-galactose (D-Gal), and non-sugar compounds like N-acetylated cysteine or acetylocholine) [12]. Among microbial ficolin-2 target structures, bacterial capsular polysaccharides, lipoteichoic acids and 1,3-β-glucans of fungal origin are mentioned [11]. It moreover recognizes elastin and DNA. Similarly to ficolin-1, ficolin-2 may contribute to the clearance of late apoptotic cells [69,70], and thus, to the maintenance of tissue homeostasis.

Several single nucleotide polymorphisms of the corresponding *FCN2* gene (involving mainly the promoter region and exon 8, which encodes for part of the fibrinogen-like domain) affecting ficolin-2 concentration and/or activity have been reported [66,71,72,73]. Until now, however, no case of total primary ficolin-2 deficiency has been found. The changes from majority allele to minority allele at positions −986 (A > G, rs3124952), −557 (A > G, rs3811140), −64 (A > C, rs78654533) and +6424 (G > T, rs7851696) were associated with a decrease in protein level concentration and/or its activity, while changes at positions −602 (G > A, rs3124953), −4 (A > G, rs17514136) and +6359 (C > T, rs17549193) had the opposite effect [71,73,74].

Ficolin-3 (also known as H-ficolin or Hakata antigen), like other ficolins recognizes acetylated sugars, but also D-fucose, L-fucose and D-galactose [12]. Although its serum concentration is the highest among lectin pathway-associated pattern recognition molecules (median in adult population is close to 20 µg/mL in adults) [49,75], few microbial ligands have been identified so far. It was, however, shown to contribute to the clearance of apoptotic cells [69,76], and to interact with ovarian cancer cells [77]. Recently, it was demonstrated that ficolin-3 and ficolin-2 may form heterocomplexes present in blood: they could have additional biological relevance compared with their parent molecules [78].

Among known *FCN3* (ficolin-3) gene polymorphisms, a frameshift mutation (+1637delC, rs28357092) has been the most widely investigated. It influences protein serum concentration significantly, leading to a rare total deficiency in variant homozygotes [79,80].

## 5. MBL-Associated Serine Proteases (MASP) and Their Related Proteins

Generally, the MASP proenzymes are single polypeptide chains, composed (like classical pathway-specific C1r and C1s) of six domains: CUB1 (C1r/C1s, urchin-epidermal, bone morphogenetic protein), EGF (epidermal growth factor), CUB2, CCP1 (complement control protein), CCP-2 and SP (serine protease, catalytic). Upon activation, the peptide bond between the CCP2 and SP domains is cleaved, resulting in the creation of heavy and light chains, linked via a disulphide bond. The CUB1-EGF-CUB2 fragment enables the dimerization of MASP molecules and complex formation with collectins or ficolins (reviewed by Kjaer et al. [81]).

### 5.1. MASP-1, MASP-3 and MAp44

MASP-1, MASP-3 and non-enzymatic MAp44 (MBL-associated protein, 44 kDA, known also as MAP-1) are products of the *MASP1/3* gene. MASP-1 cleaves C2 bound to C4b (with low efficiency), and therefore was first believed to up-regulate lectin pathway activation. However, a crucial role for MASP-2 activation has been evidenced [13]. It furthermore may contribute to coagulation cascade activation: fibrinogen, factor XIII and thrombin-activatable fibrinolysis inhibitor (TAFI) are its substrates. Moreover, its thrombin-like activity enables the cleaving of protease-activated receptor-4, being a mediator of inflammation and platelet activation (reviewed by Yongqing et al. [14] and Pihl et al. [15]) Dobo et al. [16] evidenced that another MASP-1 substrate is high-molecular-weight kininogen. This activity (like that of kallikrein) enables the release of bradykinin, a highly pro-inflammatory mediator of the contact system. Furthermore, it was demonstrated that MASP-1 contributes to the pro-inflammatory activation of endothelial cells and increases endothelial permeability [82,83,84]. Recently, MASP-1 was reported to affect the transcription of alternative pathway factor D [85].

The first described physiological substrate of MASP-3 was insulin-like growth factor-binding protein-5 (IGFPB-5). IGFPB-5 is a modulator of IGFs (factors influencing cell proliferation, differentiation, motility and survival) activity. It was also believed that, due to competition with MASP-2 for binding to the pattern-recognizing lectins, MASP-3 down-regulates lectin pathway activation (reviewed by Yongqing et al. [14] and Pihl et al. [17]) However, later reports demonstrated that the natural substrate of MASP-3 is pro-factor D, therefore it is directly involved in alternative pathway activation [18,86,87].

MAp44 (or MAP-1) has four domains in common with MASP-1 and MASP-3 (CUB1, EGF, CUB2, CCP1). An exon specific for this protein encodes additional C-terminal 17 amino acid residues. Its biological role is uncertain, however, it has been suggested to down-regulate lectin pathway activity by competitive binding to MBL with MASPs [14]. It was also evidenced to contribute to the regulation of cardiac development [88].

Several *MASP1/3* gene polymorphisms have been reported to have certain clinical associations. The SNP at position +50074 from the transcription start (G > A; rs28945068, exon 11) leads to the substitution of glycine with glutamic acid in the CCP2 domain (common for MASP-1 and -3), and therefore, was suspected to influence the function of both gene products. A/A homozygosity was suggested to be a risk factor for SIRS/sepsis [89]. Other *MASP1/3* polymorphisms have been associated with the 3MC syndrome. Most of them reside in exon 12, encoding for the protease domain of MASP-3: +1489 C > T (H497Y), +1888 T > C (C630R), +1997 G > A (G666E), +2059 G > A (G687R) [61,90]. Another SNP, +870 G > A (exon 6), leads to a stop at W290X (CUB2 domain), and affects all of the gene products [90]. Later, several other *MASP1/3* gene mutations were found within families affected by 3MC syndrome [91,92,93]. Furthermore, Haerynck et al. [94] reported the +1851 G > A (rs3821805) polymorphism (again specific for MASP-3 protease domain) to be associated with the earlier onset of chronic *Pseudomonas aeruginosa* colonization in cystic fibrosis patients. Interestingly, the substitution of guanine with adenine does not lead to an amino-acid exchange (L617L). It was speculated that such a silent mutation may affect mRNA splicing, stability, structure and protein folding [94].

### 5.2. MASP-2 and MAp19

MASP-2 and non-enzymatic MAp19 (MBL-associated protein, 19 kDa or sMAP, small MBL-associated protein) are encoded by the *MASP2* gene. MASP-2 is able to cleave C4 and C2 with high efficiency, and thus is the key enzyme for lectin pathway activation. Moreover, it is able to activate prothrombin, and thus, participate in activation of the coagulation system (reviewed by Garred et al. [19] and Pihl et al. [20]) Although another MASP-2 substrate is kininogen, its cleavage does not lead to creation of bradykinin [16]. Like MASP-1, MASP-2 was recently reported to participate in the regulation of factor D transcription [85].

MAp19 consists of two MASP-2 first domains and an additional four C-terminal amino acid residues (encoded by MAp19-specific exon) [19,20].

Several *MASP2* gene polymorphisms in various populations have been described. Some of them affect the MASP-2 serum level and/or activity. The most widely investigated, +359 A > G mutation (rs72550870), leading to an exchange of aspartic acid for glycine at position 120 (D120G; 105th residue of the mature protein, D105G), results in the rare primary MASP-2 (and MAp19) deficiency. It affects the structure of the CUB1 domain, which prohibits complexing with lectins (and thus complement activation). G/G homozygosity has been associated with recurrent infections, autoimmune manifestations and allergic symptoms, but it was found in several healthy individuals as well (reviewed by Sokolowska et al. [95]) Another possibly clinically important SNP is +1111 G > T (D371Y; rs12711521). It affects the structure of the CCP2 fragment (not present in MAp19), important for the stabilization of the protease domain, and thus, may influence the enzymatic activity of MASP-2. The G/G genotype has been demonstrated to be associated with susceptibility to HCV infection [96]. MAp19 is believed to down-regulate the lectin pathway due to competition for binding to lectins with MASP-2, but Degn et al. [97] have not confirm that property.

## 6. Associations of Lectin Pathway Components with Haematologic Malignancies

Our recent investigations revealed a higher frequency of MBL deficiency-associated genotypes (LXA/O or O/O) among multiple myeloma (MM), but not lymphoma (LYMPH) or acute myeloid leukaemia (AML) patients, compared with controls [49,98]. Furthermore, polymorphisms of the *MBL2* gene exon 4 3′-UTR seemed to influence the risk of developing lymphoma. The A/A-G/G-C/C-A/A-A/A-T/T-T/T-G/G-G/G-A/A-C/C-T/T genotype (termed “gt1”, corresponding to the following SNP: Ex4-710 A > G (rs2099902), Ex4-718 G > T (rs2099903), Ex4-845 C > T (rs2165813), Ex4-879 A > C (rs2120131), Ex4-901 A > G (rs2120132), Ex4-939 T > C (rs774307463), Ex4-1047 T > G (rs12254577), Ex4-1063 G > T (rs35768126), Ex4-1064 G > T (rs35327474), Ex4-1067 G > A (rs10824792), Ex4-1260 C > T (rs56009657), Ex4-1483 T > C (rs10082466)), was observed significantly less frequently among patients than among healthy subjects. No difference between MM and control groups was found. Although it has to be remembered that the lymphoma group included patients suffering from various diseases (Hodgkin’s lymphoma, several types of non-Hodgkin’s lymphoma) and therefore was rather heterogenous, the protective role of “gt1” cannot be excluded. The possible clinical association with *MBL2* 3′-UTR was found, for the first time, in a Caucasian (Polish) population [49].

In the context of haematopoietic stem cell transplantation (HSCT), MBL complexed with MASP was reported (in mice) to contribute to the mobilization of HSC from bone marrow to peripheral blood. MBL-MASP is able to trigger both complement and coagulation systems, cross-talking in the mobilization process [99,100]. The crucial role of MASP-1 was demonstrated in experiments with MASP-1-null mice. Interestingly, MBL-KO and MASP-1-KO animals had (after mobilization) peripheral blood cell counts and numbers of bone marrow-residing HSC which were similar to wild-type littermates [100]. It therefore was suggested that MBL-deficient patients may be poor mobilizers upon stimulation for HSCT [99,100]. On the other hand, lectin pathway activation upon the recognition of ATP (acting as danger-associated molecular pattern, DAMP) by MBL may contribute to myelodysplasia or the development of graft-versus-host disease after allogeneic haematopoietic stem cell transplantation (allo-HSCT) [101].

Both haematologic cancers themselves and their treatment including chemotherapy and/or radiotherapy result in high susceptibility to infections. Nowadays, hospital infections caused by multidrug-resistant agents (therefore especially life-threatening) constitute an increasing problem [48,102,103]. Numerous mechanisms of immune defence (phagocytosis, cellular (T cell-dependent) and humoral (B cell-dependent) response) are affected by tumour or therapy-induced immunosuppression. Additionally, central venous catheters are a common portal of entry for infecting agents. Severe iatrogenic infections in haematopoietic stem cells recipients are often caused by opportunistic pathogens [102,104]. The role of MBL in the context of infections in haematological malignancies is still controversial. Some reports suggested that MBL-deficient patients are at a higher risk of severe infections compared with MBL-sufficient individuals [105,106,107]. Molle et al. [108,109] found that MBL protects MM patients treated with melphalan and autologous haematopoietic stem cells transplantation (auto-HSCT) from severe infections, accompanied by septicaemia. Eleutherakis-Papaiakovou et al. [110] supported that conclusion to some extent, by showing the (relatively slight) association of low (<0.5 µg/mL) MBL, with an enhanced incidence of febrile episodes in a similar group of patients. Earlier, Kilpatrick et al. [111] noted more severe infections in patients with haematological malignancies when MBL serum concentration did not exceed 0.1 µg/mL. Other authors, however, found no association of MBL deficiency in severe infections after HSCT [112,113]. Later, Świerzko et al. [49] demonstrated that MBL deficiency has no influence on the incidence of hospital infections/febrile neutropenia in multiple myeloma and lymphoma patients treated with auto-HSCT. Indeed, high MBL serum concentrations before ablative chemotherapy seemed to be associated with adverse effects. However, over a 6-month period of follow-up, MBL deficiency was over-represented in the small number of patients experiencing very severe infections [49]. Furthermore, Osthoff et al. [114] based on the analysis of data from a longer follow-up, reported a similar association in recipients of allogeneic HSCT. Therefore, it seems that MBL has no (or little) protective role during the period of chemotherapy-induced cytopenia, but it may be much more important when able to act in combination with phagocytes [49]. That might explain why mannose-binding lectin is often reported to be effective based on data from longer periods of follow-up [106,115], but rather not over shorter periods [112,113]. It should be stressed, however, that Radnay et al. [116] found no greater association between MBL deficiency (<0.1 µg/mL) and the incidence of infections within one year after auto-HSCT in adult patients diagnosed with multiple myeloma or lymphoma. Recently, we reported no association of the *MBL2* gene with the risk for hospital infections or duration of fever in patients diagnosed with AML [98].

Regarding other LP-associated collectins, CL-10 and CL-11, Świerzko et al. [49] found higher serum concentrations of CL-LK (as heterocomplexes) in multiple myeloma (but not lymphoma) patients compared with controls. During a hospital stay, CL-LK levels underwent marked changes in MM patients affected by bacteraemia or febrile neutropenia, suggesting the involvement of this collectin in the immune response against some potentially life-threatening events [49].

The clinical associations of ficolins in the context of haematological malignancies have not been studied extensively. Schlapbach et al. [117] found significantly lower median ficolin-1 serum concentrations in children diagnosed with acute myeloid leukaemia (AML) and acute lymphoblastic leukaemia (ALL) (but not lymphoma) than in controls. They also reported positive correlations of ficolin-1 level with peripheral blood leukocyte counts, and proportions of both erythroid and myeloid precursors in bone marrow, but inverse correlations with leukaemic blasts in blood and bone marrow [117].

Our recent investigations [98,118] revealed markedly lower ficolin-1 concentrations in patients suffering from multiple myeloma or acute myeloid leukaemia (before chemotherapy), compared with controls. The median in the AML group was almost fivefold lower than in healthy controls (260 ng/mL vs. 1277 ng/mL). Furthermore, lower ficolin-1 before chemotherapy predicted a prolonged fever [98]. Interestingly, although ficolin-1 levels were significantly correlated with white blood cell count (WBC), hyperleukocytosis was often associated with low ficolin-1. It should be stressed, however, that no correlation with absolute neutrophil count (ANC) was noted [98].

Taking into account that ficolin-1 is synthesized in bone marrow, monocytes and granulocytes, the striking differences between patients and controls previously mentioned may reflect abnormal haematopoiesis in malignancies. In the case of MM, transient total ficolin-1 deficiency, approximately 2 weeks after conditioning chemotherapy, was noted [118].

Some *FCN1* (ficolin-1) gene polymorphisms may be associated with a higher risk of development of haematologic cancer. The genotype G/A-C/C-G/G (corresponding to SNP: −542 G > A (rs10120023), −144 C > A (rs10117466) and +6658 (rs148649884)) was found to be more common among patients diagnosed with MM than among controls [118]. A similar relationship was found for −542 G > A polymorphism and acute myeloid leukaemia [98].

Analysing the infective complications in patients, Schlapbach et al. [117] found no association between low ficolin-1 (defined as concentration < 0.5 µg/mL) and febrile neutropenia, accompanied or not by bacteraemia in children undergoing anti-cancer chemotherapy. Later, Ameye et al. [119] observed generally lower ficolin-1 concentrations in adults with various haematologic malignancies (leukaemias, lymphomas and other), undergoing chemotherapy who suffered from severe infections, in comparison with patients who did not develop such infections. In contrast, Świerzko et al. [118] found significantly higher median ficolin-1 in LYMPH patients who experienced bacteraemia compared with those who had no complications during their hospital stay. Again, as with MBL, it seems that ficolin-1 has no protective role from pathogens, but might contribute to some adverse effects within the short period after chemotherapy [118]. Recently, we found an association of A/A homozygosity for rs10117466 *FCN1* gene polymorphism (−144 C > A) with hospital infections (especially those accompanied by bacteraemia/fungaemia) in AML patients [98]. As mentioned, the A allele is associated with the higher expression of specific mRNA in monocytes and granulocytes, as well as higher serum ficolin-1.

Significantly lower serum ficolin-2 concentrations were noted in patients suffering from multiple myeloma, compared with controls [118]. Moreover, possible associations of some *FCN2* polymorphisms with haematologic cancers were found. The heterozygosity for −857 C > A and G/G homozygosity for −557 A > G (rs3811140) SNP was observed more frequently in MM compared with the C group. The latter genotype, as well as the heterozygosity for +6424 G > T (rs7851696), were also more common among LYMPH patients than among healthy individuals [118]. As mentioned, the minority alleles G (−557) and T (+6424) confer a lower concentration of the ficolin-2 protein. In contrast, median ficolin-2 serum level was higher in acute myeloid leukaemia patients, in comparison with the controls [98].

Kilpatrick et al. [111] and Ameye et al. [119] found no influence of ficolin-2 concentration on the risk of chemotherapy-related infections in adults. Later, Pana et al. [120] reported associations of GGACT, GGATG, AGACG, GGACG *FCN2* haplotypes (corresponding to the following SNP: −986 A > G, −602 G > A, −4 A > G, +6359 C > T and +6424 G > T, respectively), with prolonged episodes of febrile neutropenia and bacterial infections after chemotherapy in children diagnosed with B-cell acute lymphoblastic leukaemia. We found no impact of *FCN2* polymorphisms or serum ficolin-2 concentrations on the incidence of hospital infections in MM or LYMPH patients [118]. On the other hand, C/C homozygosity for the +6359 C > T (rs175491193) SNP (where the variant T allele is associated with relatively higher ficolin-2 level and activity) tended to be less common among patients suffering from AML who developed infections with confirmed bacteraemia/fungaemia, compared with those with no hospital infections [98].

Like ficolin-2, the median ficolin-3 concentration was found to be markedly higher in the AML group relative to healthy subjects [98]. No significant difference, however, was found in the case of multiple myeloma [118].

No impact of ficolin-3 on the incidence of infections or febrile neutropenia in adult patients with haematological malignancies was reported earlier by Kilpatrick et al. [111], Ameye et al. [119] or Islak Mutcali et al. [121] Low ficolin-3 concentration was, however, suggested to be a risk factor for febrile neutropenia (especially with bacteraemia) in paediatric cancer patients, treated with chemotherapy [122]. Our data [118] suggested that the heterozygosity for the *FCN3* gene +1637delC (rs28357092) mutation may predict elevated risks for hospital infections in patients diagnosed with lymphomas.

Data concerning associations of MASP with haematologic cancers are rather sparse. As mentioned, MASP-1 was considered to be crucial for the mobilization of stem cells from bone marrow to blood (to be collected for transplantation) [100]. Fisch et al. [123] found higher MASP-2 levels in children suffering from non-Hodgkin’s lymphomas compared with controls. Later, Świerzko et al. [49] noted no significant difference between adult patients with lymphomas (group including those diagnosed with HL and NHL) or multiple myeloma and healthy individuals. Furthermore, a chemotherapy-induced increase of MASP-2 serum concentration was observed [49].

The variant allele associated with *MASP2* gene polymorphism at position +1111 (rs1271152) seemed to be protective against diffuse large B-cell lymphoma (DLBCL) [124]. Another SNP of the same gene (rs1033638, within 3′-UTR) was considered to influence an event-free survival (EFS) in patients [125].

Relatively high MASP-2 serum concentrations were suggested to be associated with a longer EFS in children with lymphoma (especially HL) (data from retrospective study). MASP-2 was therefore supposed to be protective from infections in a longer period [126], as was later proposed also for MBL and ficolins [49,118]. In contrast, high MASP-2 determined before conditioning chemotherapy was found to be related to a higher risk of hospital (short period) infections in patients diagnosed with multiple myeloma, undergoing auto-HSCT. Interestingly, the MASP-2 level was higher in patients suffering from infections caused by Gram-positive bacteria than in those infected by Gram-negative bacteria. It inversely correlated with WBC, ANC and PLT counts, and positively with C-reactive protein (CRP) concentrations. On the other hand, a relatively high incidence of heterozygosity for the *MASP2* +359 A > G (rs72550870) mutation was noted among LYMPH patients who experienced bacteraemia [49]. The G variant allele, as previously mentioned, abolishes the formation of the collectin/ficolin-MASP-2 complex. Heterozygotes confer approximately half MASP-2 concentration in serum, compared with A/A homozygotes. It is, however, sufficient for the full activity of the MBL-dependent lectin pathway of the complement [127].

## 7. Conclusions

The literature reviewed here illustrates the complexities arising from the study of collectins, ficolins and associated serine proteases in the context of haematologic malignancies and associated infections. Their protective or harmful role could depend on multiple factors, including disease type, its stage, patient’s age, and choice of treatment.

Complement activation contributes to the elimination of oncogenic pathogens, apoptotic, necrotic or cancer cells, and thus protects host from variety of diseases. On the other hand, when uncontrolled, it may conduce to excessive or chronic inflammation, which may, in turn, facilitate the oncogenic transformation of cells, EMT or metastasis. Furthermore, both cancer itself and its treatment, including chemotherapy and/or radiotherapy, affect the expression of a variety of genes, including those specific for the complement lectin pathway. The common example of diverse relationships is MBL: its deficiency is associated with a higher risk of the development of certain malignancies, but its high concentration in serum may contribute to severe adverse effects. Moreover, it seems that (at least in some haematologic malignancies) MBL has no (or little) protective role during the period of chemotherapy-induced cytopenia, but it may be much more important when able to act in combination with phagocytes.

## Figures and Tables

**Table 1:** Collectins and ficolins—pattern recognition molecules initiating activation of complement via the lectin pathway and their associated serine proteases (mannose-binding lectin-associated serine proteases (MASP)).

Family	Protein	Ligands/Substrates	Gene and Its Chromosomal Location	Primary Sites of Expression	References
Collectins	MBL	D-Man D-GlcNAc L-Fuc	*MBL2* 10q11.2-q21	hepatocytes	[1,2,5]
	CL-10 (CL-L1)	D-Man L-Fuc D-Fuc D-Gal D-GlcNAc	*COLEC10* 8q24.12	hepatocytes	[6,7,8]
	CL-11 (CL-K1)	D-Man L-Fuc D-ManNAc	*COLEC11* 2p25.3	Kidney adrenal gland hepatocytes	[8,9,10]
Ficolins	Ficolin-1 (M-ficolin)	D-GlcNAc D-ManNAc D-GalNAc Sialic acid	*FCN1* 9q34.3	bone marrow, monocytes, neutrophils	[1,2,11]
	Ficolin-2 (L-ficolin)	D-GlcNAc D-GalNAc D-ManNAc D-Gal N-acetylated cysteine acetylocholine	*FCN2* 9q34.3	hepatocytes	[4,11,12]
	Ficolin-3 (H-ficolin)	D-GlcNAc D-GalNAc D-Gal D-Fuc L-Fuc	*FCN3* 1p36.11	hepatocytes, alveolar type II pneumocytes, ciliated bronchial cells	[1,2,11]
MASP	MASP-1	MASP-2 C2 Fibrinogen factor XIII TAFI PAR-4 kininogen	*MASP1/3* 3q27-q28	hepatocytes.	[13,14,15,16]
	MASP-3	Pro-D IGFPB-5	*MASP1/3* 3q27-q28	hepatocytes, cervix	[14,17,18]
	MASP-2	C4 C2 prothrombin kininogen	*MASP2* 1p36.3–p36.2	hepatocytes	[16,19,20]
